# Chromatin accessibility landscapes define stromal cell identities across tissues

**DOI:** 10.1038/s42003-026-09720-w

**Published:** 2026-02-25

**Authors:** Amin Nooranikhojasteh, Ghazaleh Tavallaee, Nicholas Khuu, Shu Yi Shen, Shaida Ouladan, Ayush Raman, Elias Orouji

**Affiliations:** 1 Princess Margaret Cancer Centre, University Health Network, Toronto, ON Canada; 2 Department of Pathology, McGill University, Montreal, QC Canada; 3 Epigenomics Program, Broad Institute of MIT and Harvard, Cambridge, MA USA; 4 Massachusetts General Hospital Cancer Center, Harvard Medical School, Charlestown, MA USA

**Keywords:** Epigenomics, Chromatin analysis

## Abstract

Chromatin accessibility is crucial in regulating gene expression and maintaining cellular identity. While single-cell RNA sequencing has revolutionized transcriptomic profiling, the understanding of chromatin dynamics across diverse tissues remains limited. Here, we use single-cell Assay for Transposase-Accessible Chromatin sequencing (scATAC-seq) to explore chromatin accessibility landscapes across murine tissues. We profile chromatin accessibility in 51,248 cells from nine mouse tissues, identifying 28 major cell types with distinct accessibility signatures. Our data reveal both conserved and tissue-specific cis-regulatory elements, highlighting the dynamic interplay between transcription factors and chromatin states in cell differentiation and tissue function. Motif enrichment analyses uncover transcription factor motifs driving these regulatory landscapes. Notably, we demonstrate that chromatin accessibility profiles enable tracing stromal cells, including endothelial cells, fibroblasts, and macrophages back to their tissue of origin. Using a metacell approach, we identify specific chromatin modules reflecting tissue-specific epigenomic landscapes, underscoring the role of chromatin accessibility in defining stromal cell identities. Our study provides a comprehensive atlas of chromatin accessibility across murine tissues, offering insights into the regulatory architecture that governs tissue and cell-type specificity. The ability to trace stromal cells to their tissue of origin through chromatin signatures holds important implications for diagnostics and therapeutic interventions in disease contexts.

## Abstract (web summary)

Single-cell ATAC-seq across nine mouse tissues reveals tissue-specific and shared chromatin accessibility programs, defining regulatory landscapes and cell identities through differential enhancer activity across organs.

## Introduction

Cis-regulatory elements (CREs) are regions of non-coding DNA that regulate the transcription of neighboring genes^1,2^. Different genes have specific CREs, and the presence or absence of these elements can play as a genomic switch to activate or deactivate a gene in a particular cell type or developmental stage. This specificity allows for precise control of gene expression in each tissue type^3,4^. CREs are highly conserved across species. This conservation suggests that these elements are critical for proper gene regulation and modifications in CRE sequence can have significant effects on gene expression and phenotype^5,6^.

CREs function by binding to transcription factors (TFs), and the cooperation between these TFs and their target genes creates complex regulatory circuits^7^. This interaction can either enhance or inhibit the recruitment of RNA polymerase, hence modulation of transcription, and is essential for the development, differentiation, and function of various cell types and tissues^8^. By profiling chromatin accessibility of thousands of cells from different tissues, it is possible to infer patterns in genomic regulations^9^.

Studies suggest that chromatin accessibility is a critical factor in determining cell fate and identity, and changes in chromatin accessibility can have profound effects on cell differentiation and development^10,11^. Reduced chromatin accessibility underlies irreversible disruption of a TF feedback loop, which is a key factor in the transition from self-renewal to differentiation^12^. During the process of development, chromatin accessibility works as a binary switch in a given loci to regulate cell fate^13^. Interrogation of chromatin accessibility patterns across different tissues can help understand such developmental processes. It can provide insights into how cells differentiate into various tissue types and how chromatin landscape changes during organ development.

This study aims to elucidate the chromatin accessibility landscapes across various mouse tissues at single-cell resolution using single-cell Assay of Transposase Accessible Chromatin sequencing (scATAC-seq). Here, we uncover tissue-specific chromatin accessibility patterns by comparing chromatin landscapes across different tissues. This comparison will shed light on our understanding of how chromatin accessibility contributes to tissue and cell type identity. Finally, we explored chromatin accessibility markers that can efficiently distinguish stromal cells shared in various tissues, with potential applications in disease diagnostics and therapeutic interventions.

## Materials and methods

### Frozen sample preparation for nuclei isolation

This study utilized adult C57BL6 mice aged 8–10 weeks. Tissue samples were harvested following ethical guidelines approved by the institutional ethics committee and the Research Ethics Board at the University Health Network (UHN). After collection, the tissues were washed in a petri dish with cold PBS (ThermoFisher Scientific, cat# 10010023) to remove residual blood and then dried. The tissues were cut into small pieces (approximately 50–60 mg), placed into cryovials, flash-frozen in liquid nitrogen, and stored at −80 °C until further processing.

For nuclei isolation, both pre-prepared and freshly prepared buffers were utilized. Pre-prepared buffers were filtered through a 0.22 μm PVDF filter system and stored at 4 °C. To prepare 0.75 M Tricine-KOH pH 7.8, 33.60 g of Tricine (Sigma-Aldrich, cat# T0377-100G) was dissolved in approximately 200 mL of dH2O. To this solution, 3.577 g of KOH (Millipore Sigma, cat# 1050121000), 300 μL of 1 M KOH, and approximately 1501 μL of 5 M KOH were added to adjust the pH to 7.8. The final volume was brought to 250 mL with dH2O. The 1.034x Homogenization Buffer Stable Solution was prepared by dissolving sucrose (Sigma-Aldrich, cat# S0389-500G) to a final concentration of 0.26 M in dH2O and supplementing the solution with 0.03 M KCl (Invitrogen, cat# AM9640G), 0.01 M MgCl_2_ (Sigma–Aldrich, cat# M1028-100ML), and 0.02 M Tricine-KOH pH 7.8. The diluent buffer was made by diluting 0.75 M Tricine-KOH pH 7.8 with dH_2_O to a final concentration of 120 mM, followed by supplementation with 30 mM MgCl_2_ and 150 mM KCl. The 50% Iodixanol solution was prepared by mixing OptiPrep Density Gradient Medium (60% Iodixanol, Sigma-Aldrich, cat# D1556-260ML) with the diluent buffer. Freshly prepared buffers were made on the day of the experiment and kept on ice. The 1x Homogenization Buffer Unstable Solution (HBU) was prepared by adding one tablet of cOmplete, EDTA-free Protease Inhibitor Cocktail (Sigma-Aldrich, cat# 5056489001) to the 1.034x Homogenization Buffer Stable Solution to achieve a final concentration of 1x. This solution was further supplemented with 0.001 M DTT (Sigma–Aldrich, cat# 43816-10 ML), 0.5 mM Spermidine (Millipore Sigma, cat# 85558-1G), and 0.15 mM Spermine (Sigma-Aldrich, cat# S3256-1G). The mixture was vortexed until fully dissolved, and 10% Nonidet P 40 Substitute (NP40, Sigma-Aldrich, cat# 74385-1L) was added to a final concentration of 0.3%. To prepare 30% and 40% Iodixanol solutions, the 50% Iodixanol solution was diluted with 1x HBU and mixed thoroughly.

Prior to tissue processing, a swing-bucket centrifuge was pre-chilled to 4°C, with acceleration and deceleration settings adjusted to 0. A petri dish with cover, razor blade, and forceps (cleaned with 70% ethanol) were pre-chilled on dry ice. Pestles A and B were placed in clean 15 mL Falcon tubes, and the Douncer, freshly prepared buffers, and necessary tubes (2× 2 mL Lobind, 2× 1.5 mL Lobind, 3× 1.5 mL microtubes) were pre-cooled and placed in the hood for subsequent processing steps.

### Nuclei isolation from frozen tissue

Nuclei isolation was optimized for each tissue type. A 1.5 mL Eppendorf tube was pre-weighed and chilled in a dry ice bucket for at least 5 min. Using a chilled petri dish and razor blade, a suitable piece of tissue was cut and transferred to the pre-chilled Eppendorf tube for weight measurement. Up to 30 mg of frozen mouse tissue was placed into a chilled petri dish on ice. One milliliter of cold HBU was added, and the tissue was chopped as recommended. The chopped tissue and HBU slurry were transferred to a Dounce homogenizer and incubated on ice to thaw for 5 minutes. The tissue was homogenized with up to 10 strokes of Pestle “A” on ice, followed by up to 20 strokes of Pestle “B”. The pestles were moved up and down without twisting and without pulling above the liquid-air interface.

One milliliter of the tissue slurry was aspirated using a wide-bore pipette tip. A 70 μm Flowmi cell strainer (VWR, cat# 10204-924) was pre-wet by dipping it into a 1.5 mL microtube containing HBU, and the slurry was slowly ejected into the microtube through the strainer. This step was repeated with a 40 μm Flowmi strainer (VWR, cat# 10032-802). The microtube was centrifuged at 500 g for 5 min at 4 °C. To prepare a density gradient, 400 μL of 40% OptiPrep was added to the bottom of a pre-chilled 2 mL Lobind microtube, and 400 μL of 30% OptiPrep was layered on top to form two distinct layers. After centrifugation, the supernatant (∼350–400 μL) was carefully removed without disturbing the pellet. The pellet was resuspended in 400 μL of HBU, mixed thoroughly with an equal volume of 50% OptiPrep to create 25% OptiPrep, and slowly layered on top of the density gradient. Finally, 100 μL of HBU was added on top of the OptiPrep/nuclei layer.

The sample was centrifuged at ∼3200 g for 20 min at 4 °C with the ramp and brake rates set to zero. Approximately 300 μL of liquid from the top layer was aspirated to create space for nuclei collection. Four hundred microliters of nuclei were collected from the 30–40% interface and diluted with 800 μL of HBU. To assess nuclei integrity, a microdot of SYBR Green I Nucleic Acid Gel Stain, 10,000x Concentrate (Thermo Fisher Scientific, cat# S7563) was added to 10 μL of the sample in a new 1.5 mL tube and loaded onto Countess Cell Counting Chamber Slides (Invitrogen, cat# C10228) for visualization. The isolated nuclei were pelleted by centrifugation at 500 × *g* for 5 min at 4 °C, resuspended in HBU, counted, and kept on ice until further processing.

### Optimization of nuclei preparation for each mouse tissue

As detailed above, modifications were made to the processing steps for each tissue type to optimize nuclei yield and quality, particularly up to the first centrifugation step. Generally, a chopping step was added prior to thawing tissues for lysis.

#### Lung

Due to the spongy texture of mouse lung tissue, 30 mg of frozen tissue was chopped for 5 min in a pre-chilled petri dish containing 1 mL of HBU on ice. The chopped tissue was immediately transferred to the Douncer and homogenized with 10 strokes using Pestle “A,” followed by 20 strokes with Pestle “B.” The yield for lung tissue was typically lower than other tissues, ranging from approximately 100,000 to 500,000 nuclei.

#### Pancreas

Thirty milligrams of frozen pancreas tissue was directly transferred into the pre-chilled Douncer without chopping. One milliliter of HBU was added, and the tissue was incubated on ice for 5 minutes to thaw. It was then homogenized with 10 strokes using Pestle “A,” followed by 5 strokes with Pestle “B.” The nuclei yield was approximately 1.5–2.5 million.

#### Heart

For heart tissue, 30 mg of frozen tissue was chopped on ice for 5 min and transferred to the Douncer. Homogenization was performed with 10 strokes using Pestle “A” and 12 strokes with Pestle “B.” The tissue slurry tended to form clogs and was passed through a 70 μm Flowmi filter. This filtration step, repeated as necessary, left debris on the upper surface of the 25% Iodixanol layer. The yield for heart tissue was typically 300,000 to 500,000 nuclei.

#### Kidney

Similar to the pancreas, 30 mg of frozen kidney tissue was added directly to the Douncer with 1 mL of HBU and incubated on ice for 5 min without chopping. Homogenization was performed with 10 strokes using Pestle “A” and 5 strokes with Pestle “B.” The nuclei yield was approximately 2–3 million.

#### Spleen

Spleen tissue was thawed in 1 mL of HBU on ice for 5 min in the Douncer without chopping. Homogenization involved 10 strokes with Pestle “A” and 6 strokes with Pestle “B,” yielding a high nuclei count of approximately 39 million.

#### Brain

For brain tissue, 30 mg of frozen tissue was thawed in 1 mL of HBU on ice for 5 min in the Douncer without chopping. Homogenization was performed with 10 strokes using Pestle “A” and 5–10 strokes with Pestle “B” until the slurry moved smoothly. The slurry was pipette-mixed 10 times with a wide-bore pipette tip and incubated on ice for 10 min. Approximately 300 μL of tissue slurry was passed through a 70 μm Flowmi filter at a time, using a new strainer for each pass. The nuclei yield was approximately 2–3 million.

#### Colon

Colon tissue was chopped for 3.5 min on ice, followed by a 1.5-min incubation on ice. Homogenization involved 10 strokes using Pestle “A” and 8 strokes with Pestle “B.” The nuclei yield was approximately 2–3 million.

#### Small Intestine

Small intestine tissue (30 mg) was chopped for 3 min on ice, followed by a 2-min incubation on ice. The tissue was homogenized with 10 strokes using Pestle “A” and 5 strokes with Pestle “B,” yielding approximately 2.5–3 million nuclei.

#### Liver

Liver tissue (30 mg) was thawed in 1 mL of HBU on ice for 5 min in the Douncer without chopping. Homogenization involved 10 strokes with Pestle “A” and 10 strokes with Pestle “B,” resulting in a nuclei yield of approximately 3 million.

### Single cell ATAC-seq protocol

scATAC-seq experiments were performed according to the Chromium Single Cell ATAC reagent kit v1.1 user guide (10x Genomics, CG000209 Rev F). Briefly, approximately 10,000 nuclei were mixed with ATAC Buffer (10x Genomics) and ATAC Enzyme (10x Genomics) to prepare the transposition reaction mix and incubated for 60 min at 37 °C. Transposed nuclei were mixed with a master mix and loaded onto a Chromium Next GEM Chip H along with barcoded gel beads and partitioning oil to generate single nucleus-containing GEMs using the Chromium Controller X. Upon GEM formation, gel beads were dissolved, and nucleotides were released inside individual GEMs to amplify 10x barcoded single-stranded DNA.

GEMs were incubated at 72 °C for 5 min and 98 °C for 30 s, followed by 12 cycles of 98 °C for 10 s, 59 °C for 30 s, and 72 °C for 1 min in a Bio-Rad C1000 Touch thermal cycler. Recovery Agent (10x Genomics) was added to each sample and mixed gently by inversion to break the GEMs. The pooled DNA fractions were purified using Dynabeads MyOne SILANE, washed twice with 80% ethanol, and resuspended in elution solution containing Buffer EB, 0.1% Tween 20, and 1% reducing agent B. The supernatant was mixed with SPRIselect reagent beads for a second cleanup.

Purified DNA was mixed with Sample Index PCR Mix and indexed using the Single Index Kit N Set A (10x Genomics, 3000427). Libraries were constructed under the following PCR conditions: 98 °C for 45 s, followed by 10 cycles of 98 °C for 20 s, 67 °C for 30 s, and 72 °C for 20 s, with a final extension at 72 °C for 1 minute. Libraries were subjected to a double-sided size selection with SPRIselect reagent and underwent QC on the Agilent Bioanalyzer High Sensitivity DNA chip to determine fragment size distribution. The average size of the library was quantified using the KAPA Library Quantification Kit for Illumina Platforms for sequencer clustering. The thermal cycling protocol included 95 °C for 3 min, followed by 30 cycles of 95 °C for 5 s and 67 °C for 30 s. Libraries were sequenced on an Illumina NovaSeq-6000 machine using a high-output flow cell with 50 bp paired-end reads, under the following sequencing conditions: 50 cycles (read 1), 8 cycles (i7 index), 16 cycles (i5 index), and 50 cycles (read 2).

### Single cell ATAC-seq analysis

#### Pre-processing of scATAC-seq data

Initially, FASTQ files generated from the scATAC-seq experiments were aligned to the mouse reference genome (mm10) using the STAR aligner (version 2.7.9a)^14^. Post-alignment, the aligned reads were processed using Cell Ranger ATAC-Seq v1.1.0 (10x Genomics)^15^. This tool was used to perform several steps, including de-duplication of reads to remove PCR duplicates and filtering of barcodes to retain only those associated with high-quality cells. The default parameters of Cell Ranger ATAC-Seq were utilized during this process to maintain consistency and reproducibility. Next, the processed scATAC-seq data were imported into the ArchR framework (version 1.0.1)^16^. Within ArchR, Arrow files were created from the scATAC-seq fragments using the *createArrowFiles* function. These Arrow files serve as a compact representation of the scATAC-seq data and include essential metadata and QC metrics. The fragment file was subsequently read into ArchR as a tile matrix, with a genome binning size set to 5 Kb. QC measures were applied to filter out low-quality cells, with organ-specific thresholds accounting for tissue-specific differences in TSS enrichment and fragment distributions. Specifically, cells with fewer than 1000 fragments and a transcription start site (TSS) enrichment score below 4 were excluded from further analysis, with adjustments made per tissue to ensure comparable data quality across organs. Additionally, to identify and remove potential doublets, the *addDoubletScores* function in ArchR was used. This function employs a k-nearest neighbors (KNN) approach (*K* = 10) to detect doublets based on the similarity of their accessibility profiles.

#### Dimensionality reduction, batch correction, clustering, and visualization

Following the creation of Arrow files, the next steps involved normalization and dimensionality reduction. To begin with, iterative Latent Semantic Indexing (LSI) was employed for dimensionality reduction. We conducted a total of 4 iterations of LSI, utilizing a resolution of c(0.1, 0.2, 0.4), selecting 65,000 variable features, and considering 30 dimensions while sampling 10 cells. Batch correction was performed using the Harmony algorithm implemented in ArchR via the *addHarmony* function, with a correlation cut-off parameter (corCutOff) set to 0.75^17^. Subsequently, clustering of the cells was performed using the *FindClusters* function from the Seurat package, which is integrated into ArchR through the *addClusters* function^18^. The clustering parameters were set as follows: k.param = 30, resolution = 1, and maxClusters = 60. These settings were chosen to balance between capturing fine-grained cell types and maintaining computational efficiency. To visualize the clustering results, Uniform Manifold Approximation and Projection (UMAP) was employed^19^. The parameters for UMAP embedding were adjusted to nNeighbors = 40, metric = cosine, and minDist = 0.5 to ensure distinct clustering across all samples. The entire data set clustered, and a total of 50 distinct clusters were identified and color coded in the UMAP. Subsequently, these clusters were the basis for cell type annotation. Contour plots were generated using the scDATAviz package to illustrate cell distribution across the UMAP space, with contour lines calculated using 50 bins to visualize regions of high and low cell density^20^.

#### Identification of marker genes

Initially, the resulting clusters were divided into groups based on the cells’ gene activity scores. These scores were derived using the *addGeneScoreMatrix* function in ArchR, which computes gene activity based on the accessibility of regions near gene bodies. The parameters for this function included testMethod = “wilcoxon” to perform a Wilcoxon rank-sum test and cutOff = “FDR < = 0.01 and Log2FC ≥ 1.25”. To visualize the gene activity scores, we generated dot plots illustrating the percentage of cells with active gene markers and the relative gene activity levels, highlighting the top features with the highest values for each cell type. For further refinement and visualization, we used the *addImputeWeights* function in ArchR to impute missing values, improving the accuracy of gene activity scores across cells. The markers were then visualized in the UMAP space using the *plotEmbedding* function, which projects the gene activity scores onto the UMAP coordinates. Additionally, the *plotBrowserTrack* function was utilized to visualize the markers on a genomic track plot.

#### Cell type annotation

For the annotation of cell types within the single-cell ATAC-seq datasets from nine mouse tissue types, we employed a multi-step approach integrating computational tools and manual confirmation using reference databases. The *addGeneScoreMatrix* function in ArchR was used to calculate gene activity scores, which were then used to generate DotPlots for visualizing the top marker genes for each cluster. The preliminary cell type annotation was performed by comparing the identified marker genes with known cell type markers available in Mouse Cell Atlas (MCA) database^21^. This step provided an initial classification of the cell clusters into various cell types. The annotations were further refined by integrating the scATAC-seq data with reference datasets from Tabula Muris and PanglaoDB^22,23^. We used the SingleCellExperiment and Seurat packages in R to integrate our data with these reference databases. By projecting our UMAP embeddings onto the reference datasets, we confirmed and adjusted our initial annotations to match the well-established cell types in these databases. Following the reference-based annotations, manual confirmation was performed, and any discrepancies or uncertainties were resolved through iterative reviews and adjustments.

#### Marker peaks identification

To identify marker peaks within the scATAC-seq data, we followed ArchR workflow to detect peaks associated with specific cell types.

The peak matrix was generated using the *addPeakMatrix* function in ArchR. This step creates a matrix of chromatin accessibility peaks across all cells in the dataset. The process was configured to include the ArchR project, with a ceiling value set to 4 to cap outliers.

Marker peaks were identified using the *getMarkerFeatures* function, which operates on the generated peak matrix. This function detects peaks that are differentially accessible across the predefined clusters. The dataset was divided into 28 clusters, reflecting the different cell types identified in the previous steps. The statistical significance of the identified marker peaks was assessed using the Wilcoxon rank-sum test, implemented within the *getMarkerFeatures* function. We employed a cut-off of “FDR ≤ 0.1” and “Log2FC ≥ 0.5”. The *markerHeatmap* function was used to visualize the marker peaks. Marker peaks corresponding to each cell type were extracted from the peak matrix for further analysis.

#### Pathway enrichment analysis

To gain insights into the biological significance of the identified marker peaks, we conducted gene ontology (GO) enrichment analysis for each cell type.

We first annotated the genomic regions around the TSS of marker peaks, extending from −3000 to +3000 bp. This was performed using the *annotatePeak* function from the ChIPseeker package, which leverages organism-specific and biological process databases to assign functional annotations to each peak^24^.

GO enrichment analysis was carried out using the clusterProfiler package^25^. For each cell type, marker peaks were grouped into peaksets, and GO enrichment analysis was performed to identify overrepresented GO terms. The results of the GO enrichment analysis were represented using bar plots, which highlight the top-enriched pathways.

#### Comparative analysis of cell type similarities

To visualize the similarities between different cell types, we generated a heatmap using the Jaccard similarity index and the ComplexHeatmap package^26^. This approach provides a comprehensive comparison of chromatin accessibility patterns across various cell types.

The Jaccard similarity index between data points was calculated to measure the degree of overlap between chromatin accessibility peaks across cell types. A similarity matrix was constructed from the computed Jaccard indices, where higher values indicate greater similarity between cell types.

The similarity matrix was visualized using the ComplexHeatmap package. The *Heatmap* function from the ComplexHeatmap package was used to create the heatmap, with rows and columns representing different cell types. The color intensity in the heatmap corresponds to the Jaccard similarity scores, enabling the identification of cell types with overlapping chromatin accessibility profiles.

#### Motif enrichment analysis

Motif enrichment analysis was performed to identify transcription factor binding motifs that are significantly enriched in the chromatin accessibility peaks associated with specific cell types.

The *addMotifAnnotations* function from ArchR was employed to annotate motifs in the peak regions. Default settings were used for this function, with the parameter motifSet set to “cisbp” and name set to “Motif”. Following motif annotation, motif enrichment analysis was conducted using the *peakAnnoEnrichment* function in ArchR. To obtain statistically significant results, a stringent cut-off was applied: “FDR ≤ 0.1” and “ Log2FC ≥ 0.1”.

#### Metacells identification and analysis

To identify and analyze metacells within our scATAC-seq datasets, we employed the SEACells package (v0.3.3) in a Python 3.11.7 environment^27^. This approach helps in capturing the heterogeneity within cell populations by grouping similar cells into metacells, which can then be analyzed for shared regulatory features. All metadata from the ArchR object was extracted and converted into an AnnData object using the Scanpy package (v1.8.2)^28^. Prior to metacell identification, we applied singular value decomposition (SVD) to the scATAC-seq matrix. This yielded a dimensionally reduced matrix, which was used for kernel construction. A SEACells instance was initialized with the following parameters: 90 metacells (n_SEACells), 10 waypoint eigenvalues (n_waypoint_eigs) for initialization, and a convergence tolerance (convergence_epsilon) of 1e-5.

The kernel matrix (M), capturing cell-cell similarities, was constructed based on the preprocessed data. This matrix was then visualized using a heatmap to assess the similarity structure within the dataset. Model archetypes were initialized using the *model.initialize_archetypes* function. The model underwent iterative fitting (fit), with a minimum of 10 iterations (min_iter = 10) and a maximum of 50 iterations (max_iter = 50), to refine the archetypes and assign cells to metacells. Additional refinement steps (step, 5 iterations) were performed. For similarity analysis, cell type data along with their assigned metacell numbers were extracted from the dataset containing all samples in the AnnData format. Pearson correlation was computed for each cell type and its corresponding metacells to quantify the similarity between cell types and metacell clusters.

### Reporting summary

Further information on research design is available in the Nature Portfolio Reporting Summary linked to this article.

## Results

### Identification of distinct cell types within various tissues based on their unique chromatin accessibility patterns

Single-cell transcriptomics has revolutionized our ability to profile gene expression dynamics across various tissues. Despite these advances, a detailed understanding of the interaction between transcription factors and cis-regulatory elements remains elusive. To address this gap, we produced comprehensive chromatin accessibility profiles for nine mouse tissues using single-cell ATAC sequencing.

**Fig. 1:**
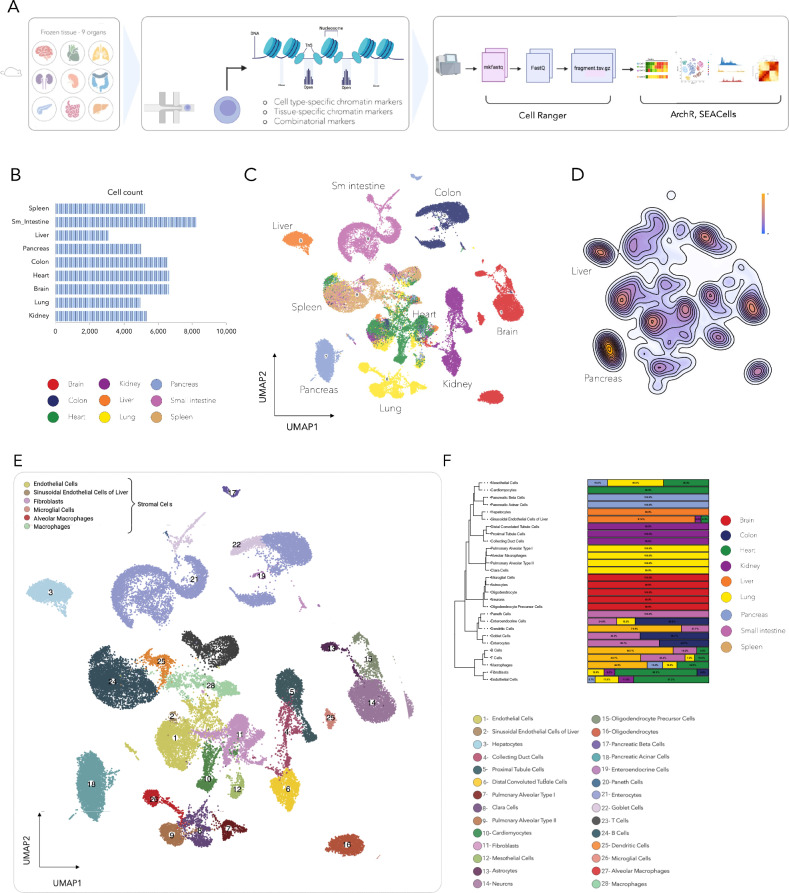
Chromatin accessibility landscapes reveal organ-specific signatures. **A** Overview of the study design, including tissue collection, nuclei isolation, single-cell ATAC-seq, and downstream analysis workflows. Single nuclei were isolated from fresh-frozen tissue using protocol optimized for each tissue type, followed by 10X genomics single cell ATAC-seq protocol. *Created with BioRender.com*. **B** Total number of cells profiled per organ across nine murine tissues. **C** UMAP visualization of chromatin accessibility profiles from nine tissues, with cells color-coded by tissue type. **D** Density plots of cell clusters within tissues, distinguishing homogeneous from heterogeneous cell populations. **E** Annotation of 28 distinct cell types across all tissues, highlighting shared and unique stromal populations. **F** Stacked bar plot showing tissue composition for each cell type.

Our analysis covered a total of 51,248 cells from frozen tissues, using a modified 10x Genomics protocol (Fig. 1A). In this study, we optimized experimental method for the nuclei isolation and processing of mouse frozen tissues in various tested organs. A detailed experimental method for tissues from which nuclei were isolated and processed is described in the methods section, and additional details are provided in the supplementary material (Supplementary Data 1 and Supplementary Information—Table 1). Quality control metrics, including cell count, nucleosome pattern, read depth, and fraction of reads overlapping peaks, were thoroughly evaluated to ensure sample integrity. The profiled cell counts per tissue varied, with the liver at 3090 and the small intestine at 8242 (Fig. 1B). All samples displayed nucleosome patterns, with the median peak read fraction of 0.42. We also assessed mean reads per cell, unique reads for each tissue, and tissue-specific TSS enrichment scores, all of which fell within optimal ranges Supplementary Figs. 1A–D). Our examination of the nine tissues revealed unique chromatin accessibility signatures for each organ (Fig. 1C and Supplementary Fig. 1E), demonstrated through UMAP visualizations where distinct cell populations were color-coded by tissue. Tissue-specific accessible regions and their associated genes were identified for each tissue (Supplementary Fig. 1F, G and Supplementary Data 2). We noted that some tissues, such as the liver and pancreas, exhibited more uniform chromatin accessibility across cells, suggesting a homogenous cell population. In contrast, organs like the heart showed greater cell type diversity (Fig. 1D). By clustering cells based on their chromatin accessibility and annotating these clusters, we identified 28 major cell types across the nine tissues. While some cell types were unique to specific tissues, stromal cells, including endothelial cells, macrophages, and fibroblasts, were common across multiple tissues (Fig. 1E). The composition of each cell type by tissue of origin, along with the number of cells annotated per cell type, is detailed in Fig. 1F.

To contextualize our findings within existing literature, we compared our 28 annotated cell types to previously published datasets, including Mouse Cell Atlas^21^, Tabula Muris^29^, and mouse sci-ATAC-seq atlas^30^. The majority of our identified cell types overlap with those reported in these comprehensive references, confirming the robustness of our annotation approach. Our contribution is not primarily the discovery of novel cell types, but rather providing detailed chromatin accessibility profiles for known cell types within a unified experimental framework. This enables direct cross-tissue comparison of chromatin states and tissue-specific regulatory elements, which has not been previously available at this scale. A comparison of cell type annotations across datasets is provided in Supplementary Information – Table 2.

These findings highlight the diversity of chromatin accessibility patterns across different tissues. The identification of shared stromal cell types across tissues provides a foundation for understanding their roles in tissue-specific contexts and inter-organ interactions.

### Interplay between tissue-specific and cell type-specific chromatin accessibility patterns

Cell type-specific chromatin accessibility refers to the chromatin configuration unique to a particular cell type, regardless of the tissue in which it resides^31^. Tissue-specific chromatin accessibility reflects the collective epigenetic state of these different cell types and is influenced by the tissue microenvironment, including factors like cell-cell interactions, extracellular matrix composition, and local signaling molecules^32^. These two levels of chromatin accessibility play crucial roles in determining gene expression profiles and cells' function within the context of a tissue.

**Fig. 2:**
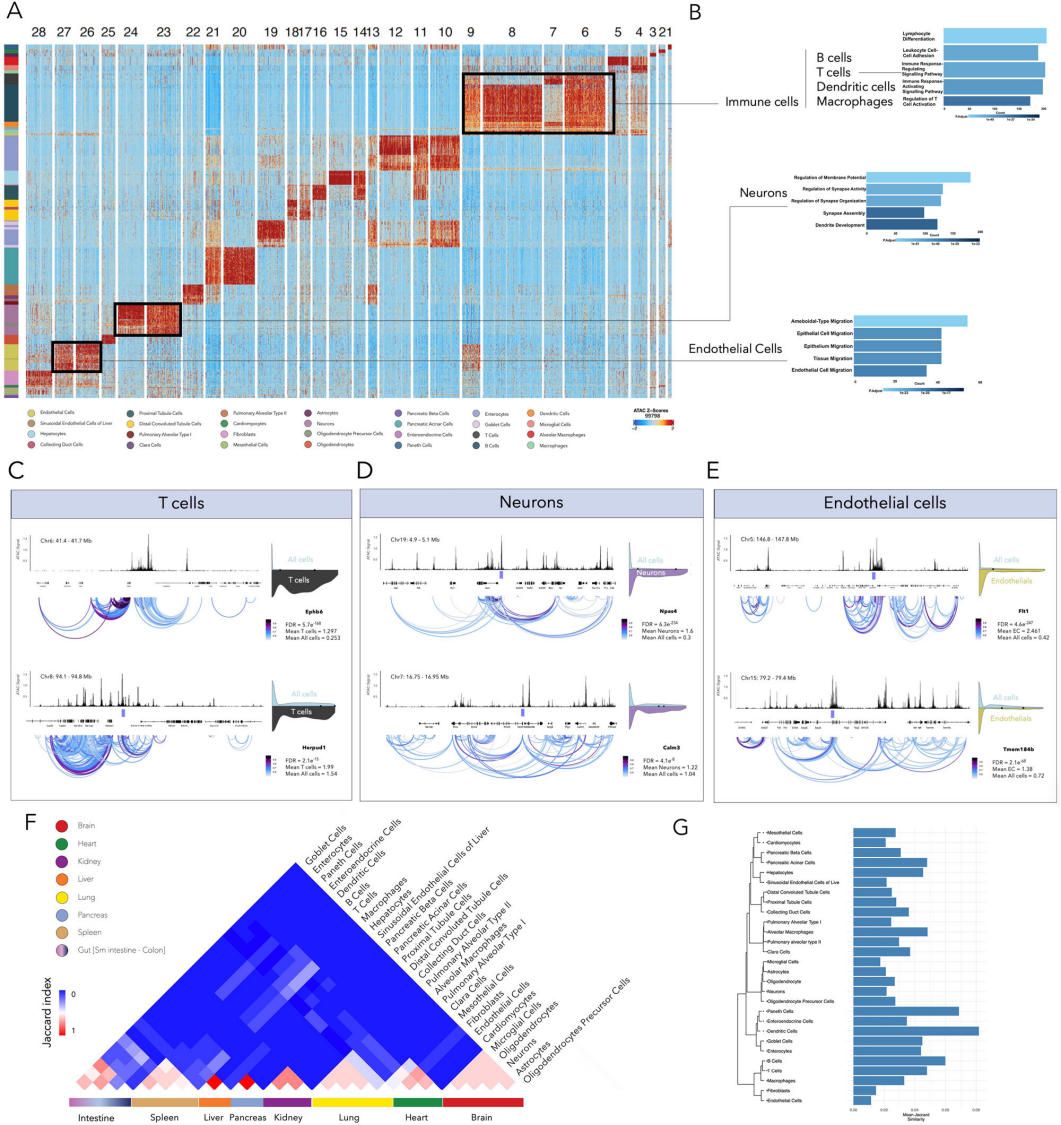
Tissue-specific and cell type-specific chromatin accessibility interplay. **A** Heatmap showing cell type-specific chromatin accessibility features. **B** Gene ontology (GO) enrichment analysis of selected cell types, illustrating pathways associated with T cells, neurons, and endothelial cells. Chromatin accessibility peaks linked to genes defining cell type function. Examples include Ephb6 and Herpud1 for T cells (**C**), *Npas4* and *Calm3* for neurons (**D**), and *Flt1* and *Tmem184b* for endothelial cells (**E**). **F** Heatmap of Jaccard similarity indices across annotated cell types, demonstrating higher accessibility similarity within tissues compared to across tissues, reflecting an overarching organ-specific signature in cell types of each tissue. **G** Bar plot showing the mean intra-cell-type Jaccard similarity of accessible chromatin regions for each annotated cell type. Stromal cell types shared across multiple organs show lower intra-cell-type similarity compared to organ-specific cell types.

To identify features that can distinguish various cell types, we performed clustering on the statistically significant chromatin accessible regions across all the tissues. Based on the analysis of highly variable peaks, we are able to identify unique chromatin accessible features in each of the annotated cell types (Fig. 2A). The number of differentially accessible peaks per cell type is provided in Supplementary Fig. 2A and Supplementary Information – Table 3. The same approach has been taken at the tissue level, and highly variable peaks were identified for each of the studied tissue types, and significant features were utilized to extrapolate expression of associated genes in these tissue types (Supplementary Fig. 1F, G and Supplementary Data 2). Gene Ontology (GO) term analysis performed on the annotated cell types could also provide further confirmation on the identity of these cell types and show key pathways that are being regulated in each of the cell types. Figure 2B represents three selected cell types and highly ranked biological pathways in those specific cells. Pathways that have been regulated in T cells are represented as part of immune cell signature within all tissues. Top-ranked upregulated pathways are also shown for the brain neurons as well as endothelial cells across multiple tissues. GO terms for the rest of cell types are shown in Supplementary Fig. 2B.

To investigate the functional relevance of chromatin accessibility patterns, we began by examining globally variable accessible regions across all cell types to identify key regulatory elements driving cell-type-specific functions. From this global analysis, we focused on the top 50 highly variable accessible regions, selecting representative regions for detailed exploration. We sought to determine how chromatin accessibility at these regions correlates with predictive gene expression patterns, as inferred from gene activity scores. By zooming into these regions, we identified significant regulatory elements and their adjacent genes, whose expression patterns highlight their roles in defining cellular identity and function. For each of the three selected cell types: T cells, neurons, and endothelial cells, we present two representative regions and the associated genes, demonstrating how chromatin accessibility influences gene expression and contributes to the biological roles of these cell types. Figure 2C shows identified highly accessible regions in T cells in the close vicinity of *Ephb6* and *Herpud1* genes, which underscores their significant roles in T cell function and the broader immune response. Ephb6 influences T cell development and function, including thymocyte maturation and receptor signaling^33^. Herpud1, critical for maintaining ER homeostasis, supports T cell activation and survival during proliferation and differentiation^34^. In neurons, highly accessible regions were identified near *Npas4* and *Calm3*, highlighting their regulatory importance. *Npas4*, located on chromosome 19, plays a critical role in neuronal gene expression and synaptic plasticity^35^, with chromatin loops suggesting extensive regulatory interactions. Calm3, on chromosome 7, is involved in calcium signaling and synaptic processes (Fig. 2D)^36^. These regions underscore key regulatory elements linked to neuronal function. Highly accessible regions near *Flt1* and *Tmem184b* were identified in endothelial cells, suggesting their roles in angiogenesis and vascular development across tissues. *Flt1*, a VEGF receptor, is essential for endothelial cell proliferation and migration^37^, while *Tmem184b* is implicated in vascular development (Fig. 2E)^38^. In Fig. 2C–E, we included gene activity score plots for nearby genes associated with the detected accessible regions, which also exhibited high gene activity scores. Supplementary Fig. 2C provides a comprehensive collection of these plots for each of the representative genomic spans, including a comparison of the mean gene activity scores for each cell type with the mean scores across all analyzed cells.

Although cell types are revealed to have a unique set of chromatin accessibility features that make them distinguishable from one another, investigating genome-wide chromatin accessibility similarities across all 28 annotated cell types shows that cell types that belong to one specific organ have a higher overall level of similarity compared to cells from different organs. Figure 2F shows a heatmap of Jaccard similarity index calculated and plotted for all 28 cell types. We observed that, with the exception of the colon and small intestine, which contain cell types with overlapping accessible chromatin features that make it challenging to distinguish between these tissues, the remaining organs studied exhibited distinct chromatin accessibility patterns. These findings highlight an additional layer of epigenetic regulation tied to tissue type. Statistical analysis of chromatin accessibility similarity within cell types, based on intra-cellular Jaccard indices and hierarchical clustering, revealed that stromal cell types shared across multiple organs exhibit lower overall accessibility similarity compared to organ-restricted cell types. Conversely, cell types with highly similar accessibility profiles clustered together, and these clusters corresponded closely to their tissue of origin. (Fig. 2G and Supplementary Fig. 2D).

### Tissue-specific chromatin accessibility shaping transcription factor dynamics and gene regulation

Accessible chromatin regions are typically free of tightly packed nucleosomes, allowing transcription factors and other regulatory proteins to bind to these genomic sites^39,40^. A unique set of transcription factors in each cell type can activate or repress transcription in their nearby genes^41^. The crosstalk between cis regulatory elements and cell-type-specific TFs can determine a unique transcriptomic signature of that particular cell type, which defines the identity and function of these cells. The interplay between accessible chromatin and TF activity is a dynamic process that allows cells to adapt to new conditions and perform different functions^42^.

**Fig. 3:**
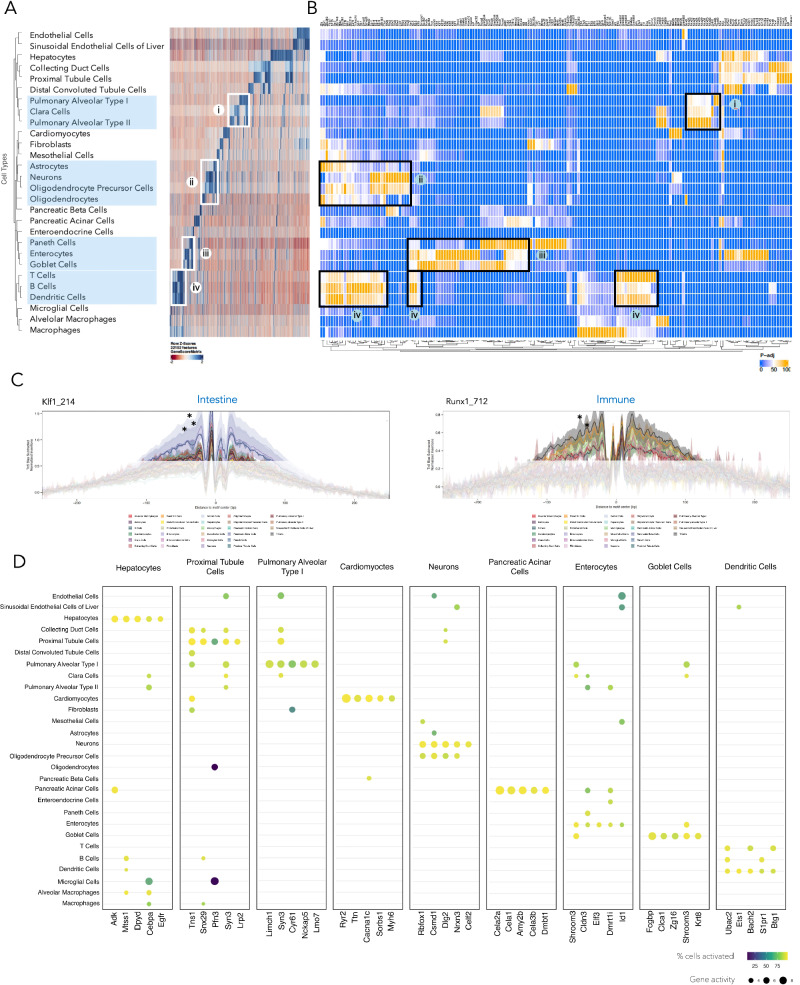
Transcription factor activity shapes cell type-specific gene regulation. **A** Highly variable chromatin-accessible regions across annotated cell types. **B** Predicted transcription factor motifs enriched in accessible regions, grouped by motif family. **C** Transcription factor footprints for *Klf1* in intestinal cell types such as goblet cells, enterocytes, and Paneth cells (indicated with asterisks) and for *Runx1* in immune cell types, such as T cells and dendritic cells (indicated with asterisks), highlighting tissue-specific motif activity. **D** Dot plot displaying the top highly regulated genes in representative annotated cell types from each of the nine investigated tissues.

Using the specific cis-regulatory signature from the chromatin accessible landscape, we identified transcription factor motifs and predicted TFs that can bind to these accessible regions. Figure 3A shows a heatmap of chromatin accessibility at highly variable marker peaks across annotated cell types, while Fig. 3B highlights the motifs of the transcription factors that bind to these CREs, regulating their target genes. This analysis allows us to explore specific cell type programs, showcasing how distinct chromatin accessibility patterns shape transcription factors and gene regulation in a tissue-specific manner.

#### Pulmonary cell program

Pulmonary cells are enriched for the Nkx family of TFs, including key members like *Nkx2-1*, which plays an essential role in lung development and function. *Nkx2-1* drives the morphogenesis that forms the lung’s structure and specifies pulmonary epithelial cell fate. These TFs regulate differentiation of lung cell types, such as type II alveolar cells, responsible for surfactant production essential for gas exchange^43^.

#### Neuronal cell program

Neuronal cells are enriched for the Rfx family of TFs, which are crucial for neuronal development, differentiation, and maintenance. These factors regulate the transformation of progenitor cells into mature neurons and the establishment of functional neural networks^44,45^.

To investigate diverse regulatory motif functions, we explore transcriptional footprinting across different tissue types. The transcription factor footprinting for the *Klf1* and *Runx1* motifs reveals distinct chromatin accessibility landscapes specific to intestinal and immune cells, underscoring the tissue-specific regulatory roles of these transcription factors (Fig. 3C).

#### Intestine cell program

The *Klf1* motif footprinting highlights elevated chromatin accessibility in intestine-related cell types, such as enterocytes and Paneth cells, with a prominent depletion at the motif center flanked by peaks of increased accessibility. This pattern suggests that *Klf1* plays a significant role in shaping the chromatin landscape in intestinal tissues, potentially influencing gene expression programs critical for gut function and development.

#### Immune cell program

The *Runx1* motif footprinting shows a clear depletion of Tn5 insertions at the motif center, indicating strong *Runx1* binding protection. This is particularly pronounced in T-cells, where the accessibility profile shows the strongest signal compared to other cell types, including dendritic cells and macrophages, suggesting a prominent functional role of *Runx1* in T-cell regulation.

Figure 3D illustrates gene activity patterns across tissues by displaying the top highly regulated genes in representative annotated cell types from each of the nine investigated tissues. The gene activity score (GAS) and the percentage of cells exhibiting activation within specific cell types are calculated and depicted. Highlighted genes represent those with both a higher GAS and broader presence across a larger population of the corresponding cell type. This analysis provides insights into the functional specialization of cell types across tissues, with highlighted genes underscoring key biological processes specific to each cell type.

Among cardiomyocytes, a significant population demonstrated GAS for key genes, including *Ryr2*, *Ttn*, *Cacna1c*, *Myh6*, and *Sorbs1*. These genes play critical roles in cardiomyocyte function, with *Ryr2* and *Cacna1c* involved in calcium signaling necessary for contraction^46^, *Ttn* providing structural support^47^, and *Myh6* being essential for contractility^48^.

In pancreatic acinar cells, genes such as *Cela2a*, *Cela1*, *Amy2b*, *Cela3b*, and *Dmbt1* exhibit high GAS in a substantial proportion of cells, emphasizing their pivotal roles in digestive enzyme production and secretion. *Cela2a*, *Cela1*, and *Cela3b* encode members of the chymotrypsin-like elastase family, which are critical for protein digestion^49^, while *Amy2b* encodes amylase, a key enzyme involved in carbohydrate digestion^50^. *Dmbt1* contributes to mucosal immunity and cellular differentiation in the pancreas^51^. The elevated GAS for these genes highlights their essential role in defining the specialized exocrine function of acinar cells in the pancreas. A comprehensive set of plots for other cell types within each organ is provided in Supplementary Fig. 3A.

### Chromatin accessibility signatures reveal tissue-specific endothelial cell identity

Chromatin accessibility markers refer to genomic regions where the chromatin state can indicate specific biological conditions or states. These markers are invaluable for understanding gene regulation mechanisms, cellular development changes, and disease progression. They offer insights into the regulatory potential of the genome and reveal early-stage epigenetic changes that might not yet be reflected in gene or protein expression.

**Fig. 4:**
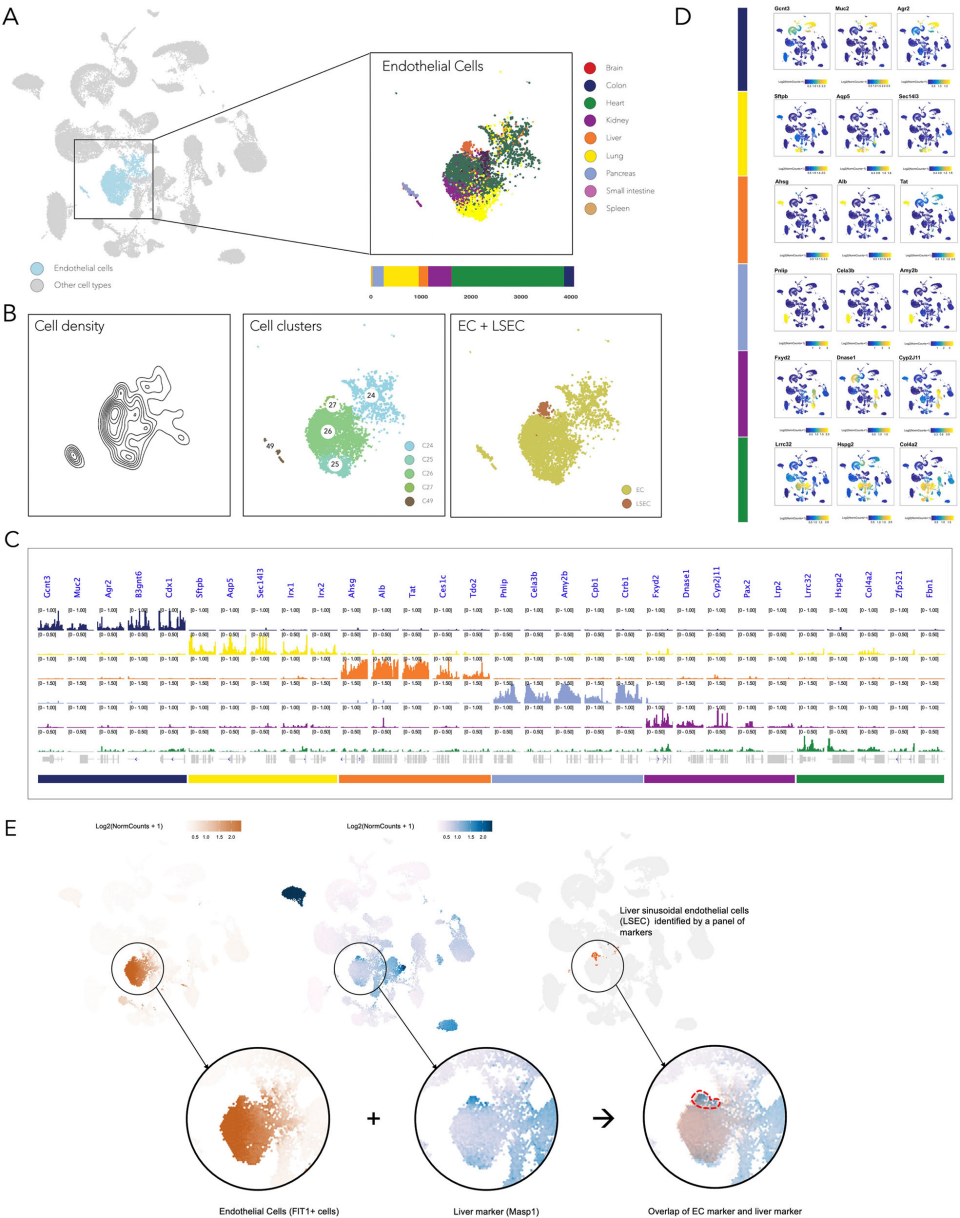
Tissue-specific endothelial cell identities revealed by chromatin accessibility. **A** UMAP visualization of endothelial cells across all tissues, with tissue distribution shown in the inset and corresponding bar plot. The heart, lung, and kidney constitute the majority of endothelial cells, respectively. **B** Cell density plot and identified cell clusters for endothelial cells. Liver sinusoidal endothelial cells (LSECs) were considered alongside endothelial cells from other tissues. **C** Genomic browser tracks showing chromatin accessibility enrichments unique to tissue-specific endothelial cells. **D** Gene activity scores for tissue-specific markers in endothelial cells, illustrating functional specialization. **E** Integration of a tissue-specific chromatin-accessible region (e.g., *Masp1*) with an endothelial cell marker (*Flt1*) in ECs to identify liver-specific endothelial cells (LSECs).

To identify cell type-specific chromatin accessibility markers, we focused on endothelial cells, which are common across multiple tissues. Figure 4A shows the UMAP of all profiled cells with highlighted endothelial cells. The right inset shows the tissue distribution within this cell type, indicating that the majority of endothelial cells are from heart tissue, followed by lung and kidney. The bar plot below the inset shows the number of cells in each tissue type. To capture the depth of the data, we included the cell density plot and identified specific cell clusters. Liver sinusoidal endothelial cells (LSECs) were considered alongside endothelial cells from other organs for further analysis (Fig. 4A, B and Supplementary Figs. 4A, B).

Within the endothelial cell population, we identified highly accessible chromatin regions with distinct patterns specific to each tissue type and linked them to nearby genes using gene activity scores (GAS). Subsequently, we evaluated the gene activity of these regions across the entire dataset. This suggests that these genomic regions and their associated genes may serve as potential markers for tissue of origin and specific markers for endothelial cells. This approach further uncovers distinct chromatin accessibility patterns in endothelial cells across different organs. Despite their shared endothelial identity, the tissue localization of these cells gives rise to unique chromatin accessibility landscapes. To this end, we have identified accessible chromatin regions associated with their activated nearby genes, which are specifically related to the tissue function in the endothelial cells of a given organ (Fig. 4C, D and Supplementary Figs. 4C–E).

#### Endothelial cells of colon

For the colon tissue, genes such as *Gcnt3*, *Muc2*, *Agr2*, *B3gnt6*, and *Cdx1* play critical roles. *Muc2* is a major component of intestinal mucus, providing a protective barrier and maintaining gut homeostasis^52^. *Agr2* is known for its involvement in mucus production and is a marker of goblet cells in the intestine^53^. *Cdx1* is a transcription factor critical for intestinal development and differentiation^54^.

#### Endothelial cells of lung

In the lung tissue, genes such as *Sftpb*, *Aqp5*, *Sec14l3*, *Irx1*, and *Irx2* are crucial. *Sftpb* is essential for the proper function of pulmonary surfactant, reducing surface tension in the lungs and preventing alveolar collapse^55^. *Sec14l3* is involved in lipid metabolism, important for surfactant production^56^. *Irx1* and *Irx2* are transcription factors important for lung development and the regulation of lung epithelial cell differentiation^57^.

#### Liver sinusoidal endothelial cells

Liver tissue is characterized by genes such as *Ahsg*, *Alb*, *Tat*, *Ces1c*, and *Tdo2*. *Alb* is the most abundant plasma protein produced by the liver, essential for maintaining osmotic pressure and transporting various substances^58^. TAT is a key enzyme in amino acid metabolism, playing a critical role in the liver’s detoxification and metabolic pathways^59^. Ces1c is involved in the hydrolysis and metabolism of various xenobiotics and drugs, highlighting its role in liver detoxification processes^60^.

#### Pancreatic endothelial cells

In pancreatic tissue, genes such as *Pnlip*, *Cela3b*, *Amy2b*, *Cpb1*, and *Ctrb1* are among the highly activated genes. Pnlip is essential for the hydrolysis of dietary fats in the digestive tract^61^. *Cela3b* is a protease that plays a key role in protein digestion^62^, and *Amy2b* is crucial for the breakdown of carbohydrates, facilitating their absorption^63^. *Ctrb1* is a precursor of chymotrypsin, an important enzyme for protein digestion^64^.

#### Renal endothelial cells

For kidney tissue, genes such as *Fxyd2*, *Dnase1*, *Cyp2j11*, *Pax2*, and *Lrp2* are essential. *Fxyd2* is involved in ion transport and maintaining electrolyte balance, which is critical for kidney function^65^. *Pax2* is essential for kidney development and the differentiation of renal epithelial cells^66^. *Lrp2*, also known as Megalin, is critical for renal reabsorption processes^67^.

#### Cardiac endothelial cells

Heart tissue involves genes such as *Lrrc32*, *Hspg2*, *Col4a2*, *Zfp521*, and *Fbn1*. *Hspg2* is a key component of the extracellular matrix, crucial for maintaining the structural integrity of cardiac tissues and facilitating cell signaling^68^. *Col4a2* is involved in the formation of the basement membrane, which supports cardiac muscle cells and maintains tissue structure^69^. *Fbn1* is essential for the structural integrity and elasticity of connective tissues in the heart^70^.

This finding could provide further evidence that certain open chromatin regions are shared among the majority of cells in a particular tissue, regardless of the cell type. Figure 4E illustrates how the integration of a tissue-specific chromatin-accessible region with an endothelial cell marker, such as *Flt1*^37^, effectively excludes non-specific cells and precisely identifies tissue-specific endothelial cells (Fig. 4E and Supplementary Information–Table 4). This approach demonstrates that combining chromatin accessibility markers with established gene biomarkers significantly enhances the identification and characterization of endothelial cells across various tissues, providing insights into their tissue-specific roles and regulatory mechanisms. We further quantified the association between chromatin accessibility and gene markers by calculating Spearman correlations. For example, accessibility at *Masp1* and *Flt1* showed a significant positive correlation (*ρ* = 0.406, *p* = 5 × 10⁻⁹) within liver endothelial cells, supporting their co-localization (Supplementary Fig. 4F, Supplementary Information–Table 4).

### Chromatin accessibility patterns in stromal cells reflect their tissue of origin

Building on our findings that chromatin accessibility signatures reveal tissue-specific endothelial cell identity, we now extend this investigation to other stromal cell types. Among the key stromal cell types, endothelial cells, fibroblasts, and macrophages play prominent roles across diverse tissues. Stromal cells, though diverse in origin and type, share several common functions across various organs. These functions are essential for maintaining tissue homeostasis, supporting organ-specific functions, and contributing to immune regulation^71^. By assessing chromatin accessibility patterns in fibroblasts and macrophages that are represented across the dataset, we aim to determine whether other stromal cell types also exhibit tissue-specific epigenomic landscapes that can serve as markers for their tissue of origin.

To investigate the potential of using chromatin accessibility as a method for tracing cells back to their tissue of origin, we employed the metacell approach. Metacells are compact groupings of highly similar cells that represent distinct cellular states while minimizing within-group variation due to technical noise. This method offers a more granular resolution than traditional clustering and is particularly effective in addressing the sparsity of single-cell data, such as that encountered in scATAC-seq datasets. Using the SEACells algorithm, metacells are defined through kernel archetypal analysis, enabling the identification of robust and comprehensive cell states across phenotypic landscapes^27^.

**Fig. 5:**
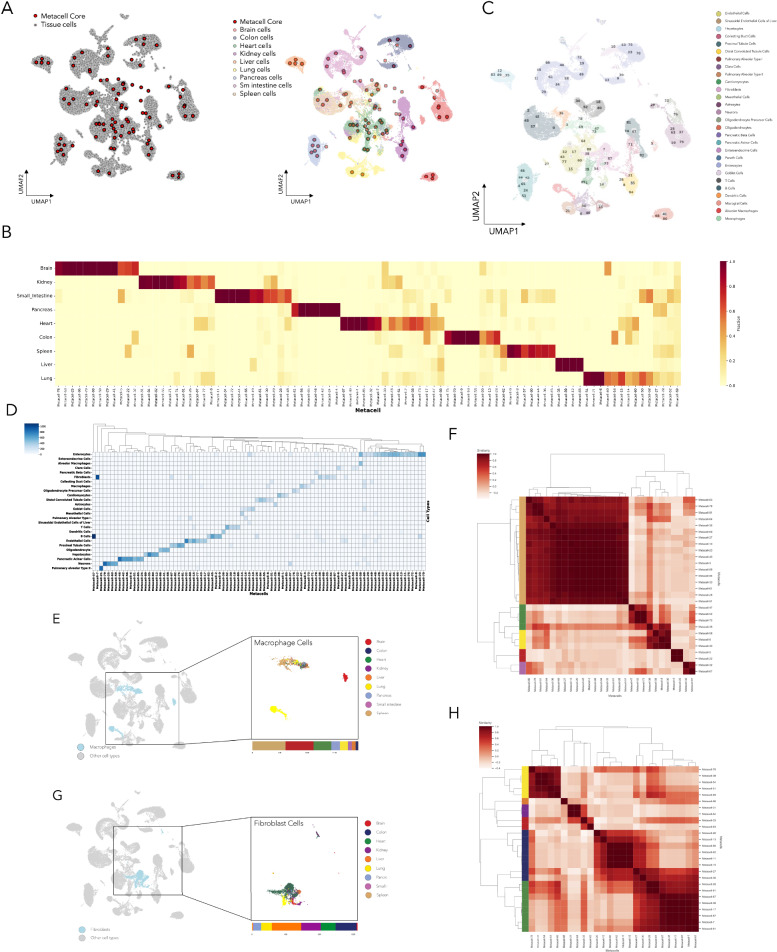
Chromatin accessibility patterns in stromal cells reflect their tissue of origin. **A** UMAP of SEACells-defined metacells (*n* = 90) across nine tissues, with tissue types color-coded. **B** Tissue composition of metacells illustrated by heatmap showing the fractional contribution of each tissue to all 90 metacells. Each column represents a metacell, and each row corresponds to a tissue of origin. Color intensity reflects the proportion of cells from a given tissue within each metacell. **C** Annotated metacells grouped by cell type and displayed on UMAP. **D** Heatmap showing metacell distribution within annotated cell types. UMAP visualization of macrophages **E** and fibroblasts **G** across all tissues, with tissue distribution shown in the insets and corresponding bar plots. Tissue-specific chromatin accessibility modules identified in macrophages (**F**) and fibroblasts (**H**), visualized with heatmaps of Jaccard similarity indices. Distinct chromatin accessibility modules can be recognized on the heatmap, which significantly overlap with the metacells of a specific organ. Tissue types are color-coded on the left side of the heatmaps.

Applying this approach, we identified 90 distinct metacells across the dataset, capturing coherent subgroups within broader cell types (Fig. 5A). To characterize their tissue origins, we quantified the fractional contribution of each tissue within every metacell and visualized these proportions as a tissue-composition heatmap in Fig. 5B and Supplementary Fig. 5A. This representation provides a detailed view of tissue enrichment patterns and reveals that most metacells display strong tissue-specific composition. To further examine metacell-specific chromatin patterns, we explored metacells within each of the 28 annotated cell types. Figure 5C presents a UMAP where metacells corresponding to distinct cell types are clearly demarcated, while Fig. 5D provides a heatmap showing the distribution of metacells and their relative dominance within each cell type.

We used macrophages and fibroblasts as two cell types that can be found in multiple different tissue types. We implemented and identified metacells to explore intra-cell-type homogeneity and find chromatin accessibility similarities within these cell types of each particular tissue. This approach resulted in the identification of particular chromatin accessibility modules that accurately associate with the tissue of origin.

Macrophages are predominantly observed in the spleen but are also found in other tissues. Figure 5E highlights the macrophage distribution across tissues, with the inset bar plot showing the number of macrophages identified in each tissue type (Supplementary Fig. 5B). By analyzing metacells derived from the macrophage subpopulation, we observed chromatin accessibility modules that displayed significant tissue-specific overlaps. Heatmap in Fig. 5F shows identified chromatin accessibility modules that overlap significantly with the tissue of origin of metacells (Supplementary Fig. 5C). These tissue-specific chromatin accessibility patterns are consistent with published studies demonstrating distinct epigenetic states in tissue-resident macrophages across different organs^72,73^.

Similarly, fibroblasts, another key stromal cell type, were analyzed for tissue-specific chromatin patterns. Figure 5G highlights the fibroblast distribution across the dataset, with fibroblasts shown in blue and tissue types marked in corresponding colors. Metacell-based analysis revealed chromatin accessibility modules that were strongly associated with the tissue of origin, comparable to the findings for macrophages (Fig. 5H).

This expanded analysis enables us to explore the broader utility of chromatin accessibility patterns in defining tissue identity across key stromal cell types, complementing the findings for endothelial cells discussed earlier.

## Discussion

Our study leveraged single-cell ATAC-seq to generate high-resolution chromatin accessibility profiles across nine mouse organs. Previous cell atlases, such as Tabula Muris, the Mouse Cell Atlas, and the sci-ATAC mouse atlas, established the major cellular constituents of murine organs but were not designed to resolve how shared stromal populations differ epigenetically across tissues. By contrast, our study provides a unified chromatin accessibility atlas across nine organs, revealing that endothelial cells, fibroblasts, and macrophages, although present in multiple tissues, retain tissue-of-origin chromatin signatures that are sufficiently robust to trace these populations back to their native organ. This extends beyond prior work by showing that shared stromal cell types exhibit distinct regulatory landscapes reflective of their microenvironment. A foundational precedent for mapping chromatin accessibility across multiple tissues is provided by Cusanovich et al., who pioneered a combinatorial indexing approach to generate one of the first large-scale single-cell chromatin accessibility atlases. Their work revealed extensive cell-type–specific regulatory elements and demonstrated how chromatin accessibility encodes cellular identity across diverse tissue contexts. Our study builds on this foundation by providing higher-resolution, metacell-based regulatory structure and by directly comparing stromal populations across organs. Unlike earlier atlases that primarily resolved broad cell classes, our dataset delineates tissue-adapted chromatin accessibility patterns within shared stromal cell types, offering deeper granularity into how microenvironmental context shapes the regulatory genome^30^. Moreover, by integrating tissue-specific accessible regions with canonical cell-type markers, we identify combinatorial chromatin features that enable precise localization of stromal cells, a level of resolution that cannot be achieved through transcriptomic markers alone. Finally, the depth and breadth of our dataset, capturing stromal populations across nine organs, provide a comprehensive resource for comparative epigenomic analyses and establish a framework for understanding how chromatin accessibility encodes both cellular identity and tissue context. By profiling over 51,000 cells, we uncovered distinct chromatin accessibility signatures that are both cell-type- and tissue-specific, reinforcing the role of chromatin accessibility in defining cellular identity.

### Tissue-specific chromatin accessibility and its implications

One of the key findings of this study is the extent to which chromatin accessibility is shaped not just by cell type but also by the tissue microenvironment. While previous studies have primarily focused on chromatin accessibility at the single-cell level within specific tissues, our multi-organ approach allowed us to examine how chromatin landscapes vary across different physiological contexts. Notably, our data show that even widely distributed stromal cell populations, including endothelial cells, fibroblasts, and macrophages, exhibit tissue-specific chromatin accessibility patterns, suggesting that these cells are epigenetically tuned to their local microenvironments^74,75^. This observation is consistent with prior endothelial cell atlases, such as the cross-tissue single-cell RNA-seq atlas, which demonstrated that endothelial cells adopt highly tissue-adapted transcriptional programs across organs. Our chromatin accessibility data extend this principle to the epigenomic level, showing that the regulatory landscapes of endothelial cells similarly reflect their tissue-specific microenvironment^76^.

Interestingly, despite these broad differences, certain chromatin accessibility modules were conserved across multiple tissues, particularly in immune-related cells. For example, T cells and macrophages displayed shared regulatory elements regardless of their tissue of origin, pointing to a core chromatin program that governs immune function. However, subtle variations in accessibility profiles still allowed us to distinguish tissue-resident immune cells, reinforcing the idea that even highly plastic cell types maintain some level of tissue specificity.

### Chromatin accessibility as a tool for tracing tissue of origin

One of the most striking applications of our findings is the potential for using chromatin accessibility as a marker of tissue origin. Our metacell analysis demonstrates that the tissue microenvironment shapes stromal cell chromatin accessibility, as evidenced by tissue-specific clustering patterns (Fig. 5F, H) and the identification of distinct chromatin accessibility modules linked to tissue of origin. Across macrophages and fibroblasts, we identified 23,772 and 6001 tissue-specific accessible peaks, respectively, while other cell types showed similarly extensive regulation (e.g., 48,757 in enterocytes, 23,477 in T cells, and 34,520 in B cells) **(**Supplementary Information–Table 4), providing a quantitative measure of loci influenced by tissue context. Given that chromatin states are often retained even when cells migrate or undergo transformation^77,78^, our findings suggest that chromatin accessibility profiling could be a powerful tool for tracing the origins of tumor cells in metastatic disease.

Moreover, the ability to distinguish endothelial cells, fibroblasts, and macrophages based on tissue-specific chromatin accessibility has broad implications for regenerative medicine. Understanding the chromatin landscape of these stromal cell populations may provide insights into how tissues maintain homeostasis and respond to injury, potentially informing strategies for tissue engineering and cell-based therapies.

### Regulatory elements and transcription factor networks

Through motif enrichment analysis and transcription factor footprinting, we identified key transcription factors driving tissue-specific chromatin accessibility landscapes. This supports the concept of transcription factor networks orchestrating complex gene regulatory circuits, as described in previous research^79–81^. Notably, different organs exhibited enrichment for distinct transcription factor motifs that are critical for organ-specific functions. For example, lung-resident cells were enriched for Nkx-family transcription factors, which play essential roles in pulmonary development and epithelial cell differentiation. Similarly, endothelial cells across tissues exhibited differential accessibility at VEGF-associated regulatory elements, which likely contribute to the specialized functions of blood vessels in different organs.

This level of granularity in chromatin accessibility provides important insights into how different tissues establish and maintain their cellular identities. It also highlights the complexity of transcriptional regulation, where a combination of broadly shared and tissue-specific regulatory elements works in concert to define cell fate.

### Translational implications of chromatin accessibility

Our study also demonstrated that cell type-specific chromatin accessibility can be used to identify markers for various physiological and pathological states. The combinatorial chromatin accessibility markers provide a powerful approach for distinguishing cell types, which can be particularly useful for diagnostic and therapeutic applications.

In diseases like cancer, despite efforts for targeted therapies, many treatments can impact chromatin accessibility in a wide range of organs^82,83^. Identification of organ-specific accessible chromatin regions enables mapping of tissue-restricted regulatory elements and transcription factor networks. By targeting these lineage-specific enhancers rather than ubiquitously expressed factors, therapeutic strategies can be designed to selectively modulate gene expression in the tissue of origin while minimizing off-target effects in other organs, thereby reducing treatment-related adverse effects.

In conclusion, our study provides a detailed map of chromatin accessibility across multiple mouse tissues at single-cell resolution, highlighting the interplay between chromatin state, gene regulation, and cellular identity. Different cell types, even within the same organ, can have distinct chromatin accessibility patterns, due to expressing different sets of genes and having various functional roles. However, each tissue in the body has a unique function and cellular composition, which can be reflected in its overall chromatin accessibility pattern. This collective pattern explains unique tissue-specific function and may have been derived from shared regulatory mechanisms across the tissue. Thus, while there are significant variations across different cell types within a specific organ, there may be overarching themes in chromatin accessibility within that specific organ. Identification of such themes could serve as a resource for chromatin accessibility landscape in normal tissues and help investigators in the field to utilize it for comparative analysis with pathological states. Future studies can build on this work by exploring chromatin accessibility in pathological states and leveraging these insights for therapeutic interventions.

## Supplementary information

Supplementary Information

Supplementary Data 1

Supplementary Data 2

Description of Additional Supplementary Files

Reporting Summary

## Data Availability

The data generated in this study have been deposited in the Gene Expression Omnibus (GEO) under the accession number GSE286329 and are publicly available. The dataset includes raw single-cell ATAC-seq data as fragment files, processed peak files in BED format, and chromatin accessibility matrix files.

## Code availability

All original code has been deposited on Zenodo (10.5281/zenodo.18227472) and GitHub (https://github.com/OroujiLab/Mouse_Tissues_scATAC) and is publicly available.
