# Supplementary material for: Chromatin accessibility landscapes define stromal cell identities across tissues
Source: Commun Biol. 2026 Feb 25;9:480. doi: 10.1038/s42003-026-09720-w (PMC13043806; doi:10.1038/s42003-026-09720-w)
Supplement: Supplementary file 1 — Supplementary Information [file 42003_2026_9720_MOESM1_ESM.pdf]

Supplementary Figure 1.

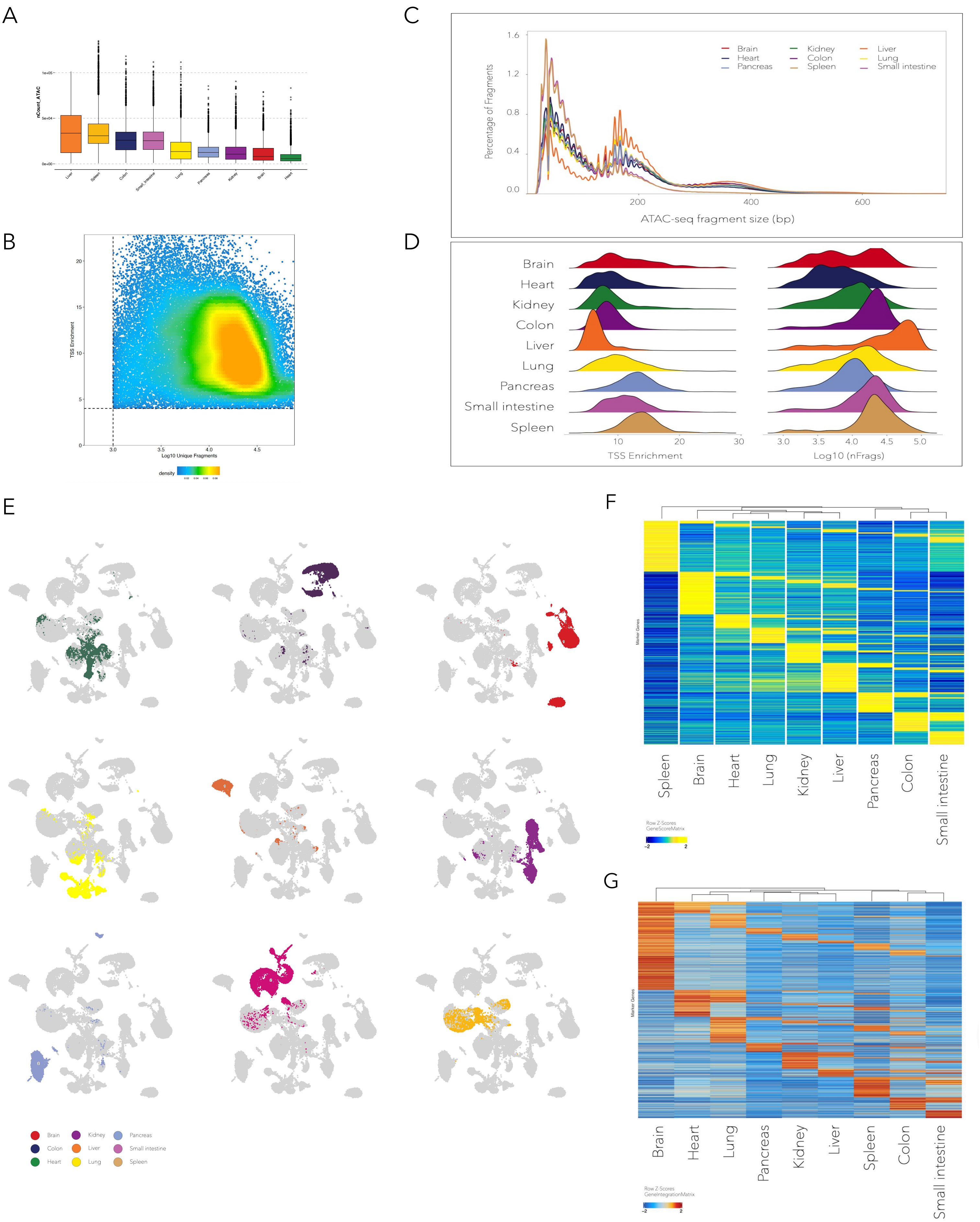

Supplementary Figure 2.

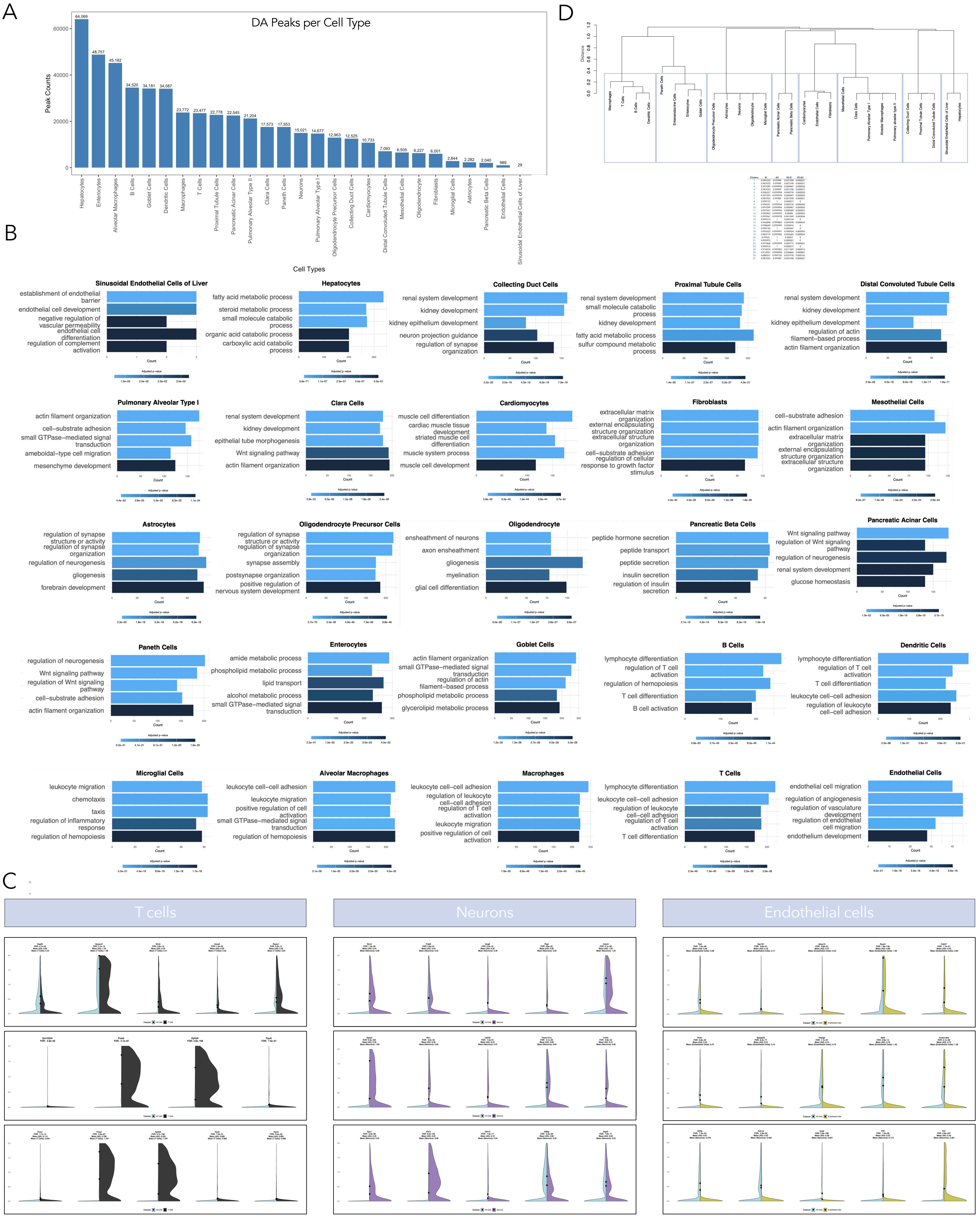

Supplementary Figure 3.

A

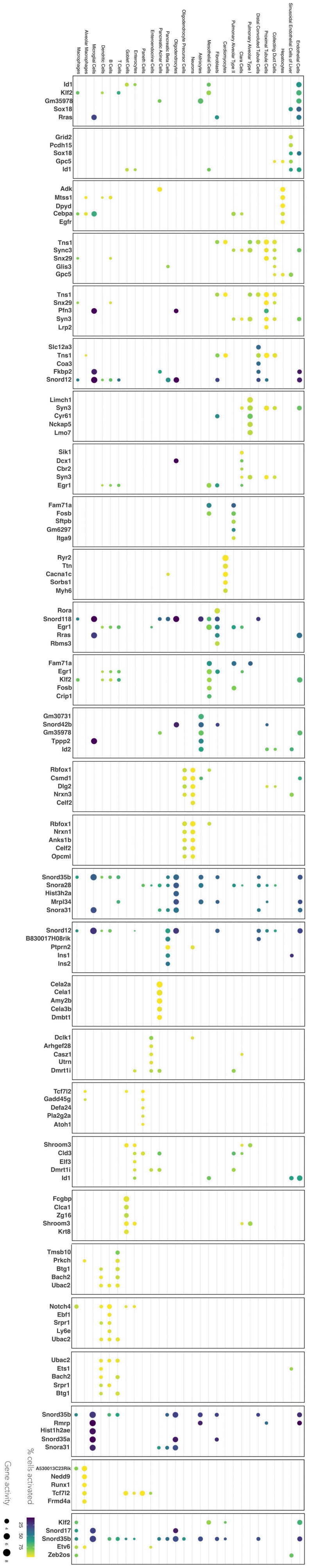

Supplementary Figure 4.

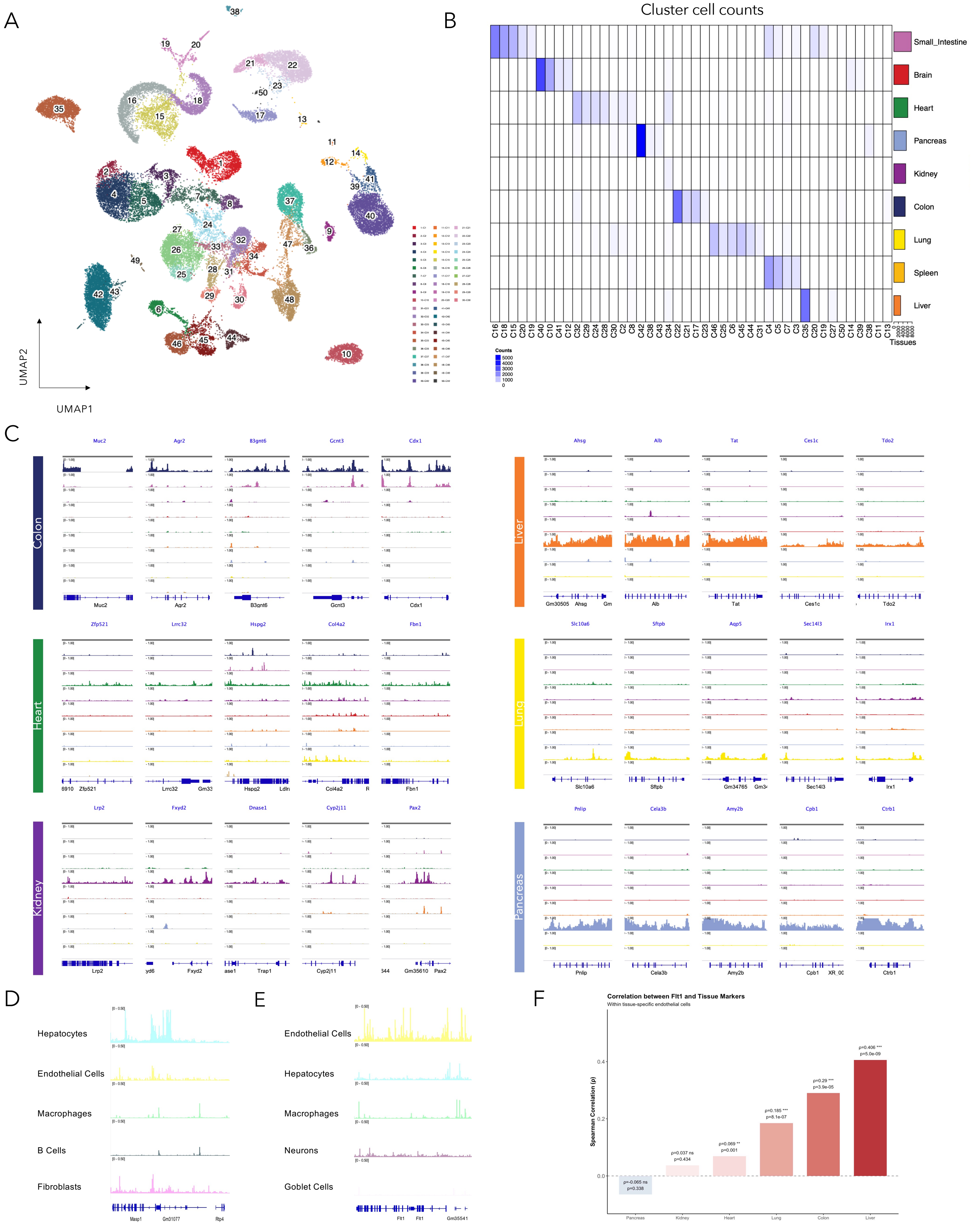

Supplementary Figure 5.

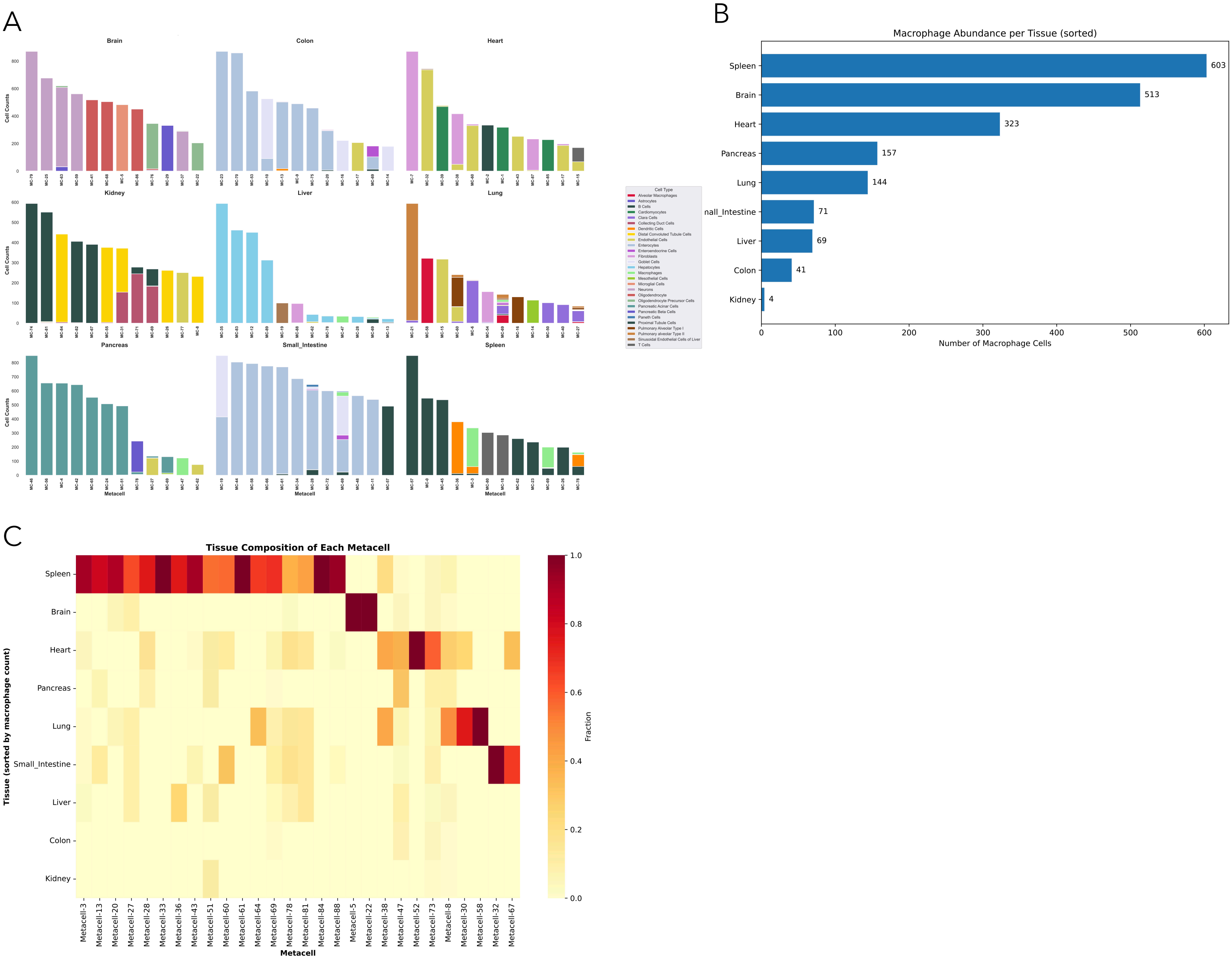

Supplementary Figure Legends

Supplementary Figure 1. Quality control metrics for scATAC-seq datasets.
(A) Distribution of total fragment counts per cell across nine tissues, represented as box plots. (B) Scatter plot depicting the density of cells based on transcription start site (TSS) enrichment and the log10-transformed number of unique fragments. (C) ATAC-seq fragment size distribution profiles for all tissues, highlighting nucleosome-free, mono-nucleosome, and di-nucleosome fragments. (D) Ridge plots showing tissue-specific distributions of TSS enrichment (left) and log10-transformed fragment counts (nFrags, right). (E) UMAP visualization showing tissue-specific clustering of scATAC-seq profiles. Each panel highlights cells from a single tissue (brain, kidney, pancreas, colon, liver, heart, lung, spleen, and small intestine) in color, with cells from other tissues shown in gray. (F) Heatmap illustrating tissue-specific chromatin accessibility derived from the GeneScoreMatrix. The GeneScoreMatrix quantifies accessibility at gene promoter regions, providing a direct measure of chromatin openness linked to gene regulation. Each row represents a gene, and each column corresponds to a tissue. Row Z-scores of accessibility are computed to standardize and visualize tissue-specific regulatory patterns, with hierarchical clustering revealing distinct clusters of tissue-enriched accessible regions. (G) Heatmap displaying tissue-specific gene regulatory programs derived from the GeneIntegrationMatrix. Rows represent genes with tissue-specific regulatory associations, while columns correspond to tissues.

Supplementary Figure 2. Tissue-specific chromatin accessibility patterns across organs.
(A) Bar plot showing the count of differentially accessible (DA) chromatin peaks identified for each of the 28 annotated cell types ($FDR \leq 0.1$, $Log2FC \geq 0.5$).
(B) GO enrichment analysis for the additional cell types, detailing tissue-specific biological processes. (C) Gene activity score comparisons for marker genes in T cells, neurons, and endothelial cells. Violin plots display the distribution of gene activity scores for representative marker genes (rows) across the specific cell type (colored) versus all other cells (gray). Each panel corresponds to a genomic region shown in Figure 2C-E, demonstrating quantitative enrichment of marker gene activity in the corresponding cell type. (D) Dendrogram clustering displaying AU (Approximately Unbiased) p-value generated using pvclust, an R package for hierarchical clustering with p-values. The AU p-value is computed by multiscale bootstrap resampling. Additionally, the standard inclusion probability (SI) values and their corresponding standard errors are provided.

Supplementary Figure 3. Tissue- and cell type-specific gene regulation across 28 annotated cell types. (A) Dot plot illustrating the top highly regulated genes across all 28 annotated cell types from the nine investigated tissues, highlighting key tissue-specific and cell type-specific gene regulatory programs.

Supplementary Figure 4. Annotation of clusters and identification of chromatin markers.
(A) Pre-annotation UMAP of all cells into 50 distinct clusters using Leiden clustering. Subsequently, these clusters were the basis for annotation of the resulting 28 cell types. (B) Cell counts within each cluster. (C) Genomic browser tracks of representative marker peaks showing chromatin accessibility enrichments unique to tissue-specific endothelial cells. (D) Comparison of tissue-specific versus cell-type-specific chromatin accessibility. Masp1 locus shows accessibility across multiple cell types within liver (hepatocytes, endothelial cells, macrophages) but not in

endothelial cells from other organs, demonstrating tissue-context chromatin signals. (E) Flt1 locus shows endothelial-specific accessibility across all tissues, with minimal signal in non-endothelial cells. (F) Bar plot showing Spearman correlation coefficients between the pan-endothelial marker Flt1 and tissue-specific markers (Liver: Masp1, Colon: Muc2, Lung: Sftpb, Heart: Hspg2, Kidney: Fxyd2 and Pancreas: Pnlip) calculated within tissue-resident endothelial cells. Bars are colored by correlation strength (red = positive, blue = negative). Significance: ***p<0.001, **p<0.01, *p<0.05, ns = not significant.

Supplementary Figure 5. Metacell composition and macrophage distribution.
(A) Distribution of metacells in each tissue, represented as a bar plot. (B) Number of macrophage cells per tissue. (C) Tissue composition of the 28 macrophage-enriched metacells analyzed in Figure 5F-H, demonstrating tissue-specific clustering within the macrophage population.

Table 1. Cell type by tissue cross-tabulation.

	Brain	Colon	Heart	Kidney	Liver	Lung	Pancreas	Small intestine	Spleen
**Alveolar Macrophages**	0	0	0	0	0	678	0	0	0
**Astrocytes**	467	0	0	0	0	1	0	0	0
**B Cells**	3	44	400	4	58	116	71	788	2846
**Cardiomyocytes**	2	24	1030	2	0	0	0	5	0
**Clara Cells**	0	0	0	0	0	929	1	0	0
**Collecting Duct Cells**	9	0	2	781	0	0	0	0	0
**Dendritic Cells**	0	15	9	0	0	1	0	140	481
**Distal Convoluted Tubule Cells**	0	0	0	1523	0	0	1	0	0
**Endothelial Cells**	6	195	2242	453	53	700	221	8	32
**Enterocytes**	0	3592	6	1	1	0	0	5215	3
**Enteroendocrine Cells**	0	76	0	0	0	19	0	30	0
**Fibroblasts**	91	250	1751	233	126	348	92	9	8
**Goblet Cells**	0	679	0	0	0	0	0	518	0
**Hepatocytes**	0	0	0	0	2477	1	1	0	0
**Macrophages**	23	41	323	4	69	144	157	71	603
**Mesothelial Cells**	0	3	168	1	0	208	74	8	0
**Microglial Cells**	490	0	0	0	0	0	0	0	0
**Neurons**	3254	0	0	0	0	3	0	0	0
**Oligodendrocyte**	1504	0	0	0	0	0	0	0	0
**Oligodendrocyte Precursor Cells**	628	12	0	0	0	1	0	0	0
**Pancreatic Acinar Cells**	0	0	1	0	0	0	4605	0	1
**Pancreatic Beta Cells**	0	0	0	0	0	0	223	0	0
**Paneth Cells**	0	0	0	0	0	0	0	298	0
**Proximal Tubule Cells**	0	0	0	2185	0	0	1	0	0
**Pulmonary Alveolar Type I**	0	0	0	0	0	540	0	0	0
**Pulmonary Alveolar Type II**	0	0	0	0	0	1052	0	0	0
**Sinusoidal Endothelial Cells of Liver**	0	0	10	8	139	0	1	0	1
**T Cells**	3	48	177	2	48	118	37	546	656

Table 2. Comparison of the number of identified cell types resolved by scATAC-seq in our study and those reported in two major scRNA-seq atlases.

Study	Identified cell types	Modality	Citation
**Mouse Cell Atlas (MCA)**	46 (for 9 organs)	single cell RNA-seq	Han, X. et al. *Cell* **172**, 1091–1107.e17 (2018).
**Tabula Muris**	67 (for 9 organs)	single cell RNA-seq	Tabula Muris Consortium *Nature* **562**, 367–372 (2018).
**Our study**	28	single cell ATAC-seq	

Table 3. The number of differentially accessible (DA) chromatin peaks identified for each cell type (FDR ≤ 0.1, Log2FC ≥ 0.5).

Cell type	n_DA_peaks
**Hepatocytes**	64069
**Enterocytes**	48757
**Alveolar Macrophages**	45182
**B Cells**	34520
**Goblet Cells**	34181
**Dendritic Cells**	34087
**Macrophages**	23772
**T Cells**	23477
**Proximal Tubule Cells**	22778
**Pancreatic Acinar Cells**	22545
**Pulmonary Alveolar Type II**	21204
**Clara Cells**	17573
**Paneth Cells**	17553
**Neurons**	15021
**Pulmonary Alveolar Type I**	14677
**Oligodendrocyte Precursor Cells**	12963
**Collecting Duct Cells**	12525
**Cardiomyocytes**	10733
**Distal Convoluted Tubule Cells**	7093
**Mesothelial Cells**	6505
**Oligodendrocyte**	6227
**Fibroblasts**	6001
**Microglial Cells**	2844
**Astrocytes**	2282
**Pancreatic Beta Cells**	2040
**Endothelial Cells**	989
**Sinusoidal Endothelial Cells of Liver**	29

Table 4. Correlation analysis between expression of the endothelial marker Flt1 and chromatin accessibility markers for each tissue, provided as supplementary data to Fig. 4E.

Tissue	Marker1	Marker2	Correlation	P value	N cells
**Liver**	Flt1	Masp1	0.406	5.03E-09	192
**Lung**	Flt1	Sftpb	0.185	8.14E-07	700
**Heart**	Flt1	Hspg2	0.069	1.09E-03	2252
**Kidney**	Flt1	Fxyd2	0.037	4.34E-01	461
**Colon**	Flt1	Muc2	0.29	3.88E-05	195
**Pancreas**	Flt1	Pnlip	-0.065	3.38E-01	222
