# Supplementary material for: Chromatin accessibility landscapes define stromal cell identities across tissues
Source: Commun Biol. 2026 Feb 25;9:480. doi: 10.1038/s42003-026-09720-w (PMC13043806; doi:10.1038/s42003-026-09720-w)
Supplement: Supplementary file 4 — Description of Additional Supplementary Files [file 42003_2026_9720_MOESM4_ESM.docx]

**Description of Additional Supplementary Files**

**File name:** Supplementary Data 1

**Description:** Reagents, buffers, and materials used for tissue preparation and nuclei isolation.

**File name:** Supplementary Data 2

**Description:** Tissue-specific accessible chromatin regions and associated gene annotations.
